# (*E*)-*N*-[2-(9-Fluorenyl­idene)-3a,5,7-tri­methyl-3,3a-dihydro-2*H*-indol-3-yl­idene]-2,4,6-trimethyl­aniline

**DOI:** 10.1107/S1600536808001657

**Published:** 2008-01-23

**Authors:** Yoshiyuki Mizuhata, Norihiro Tokitoh

**Affiliations:** a Institute for Chemical Research, Kyoto University, Gokasho, Uji, Kyoto 611-0011, Japan

## Abstract

The title compound, C_33_H_30_N_2_, has an *E* configuration at the imine double bond. The angle between the least-squares planes of the imine C=N—C group and the benzene ring of the 2,4,6-trimethylphenyl substituent is 85.38 (11)°. The crystal structure is sustained mainly by inter­molecular π–π inter­actions (3.510 Å) between the two fluorene rings and some C—H⋯π inter­actions.

## Related literature

For related literature, see: Döpp *et al.* (1985 ▶); Gerlach & Arnold (1997 ▶); Miyata *et al.* (1999 ▶); Mizuhata *et al.* (2005 ▶); Murakami *et al.* (1996 ▶); Shimizu *et al.* (1991 ▶).
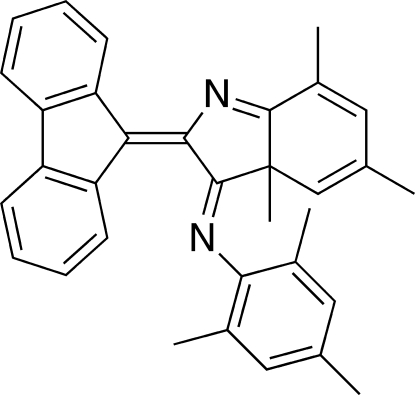


## Experimental

### 

#### Crystal data

C_33_H_30_N_2_
*M* *_r_* = 454.59
Monoclinic, 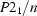

*a* = 10.2810 (2) Å
*b* = 11.2727 (3) Å
*c* = 21.6598 (5) Å
β = 102.5953 (16)°
*V* = 2449.84 (10) Å^3^
*Z* = 4
Mo *K*α radiation
μ = 0.07 mm^−1^
*T* = 103 (2) K
0.20 × 0.20 × 0.05 mm

#### Data collection

Rigaku Mercury diffractometer
Absorption correction: multi-scan (*REQAB*; Jacobson, 1998 ▶) *T*_min_ = 0.986, *T*_max_ = 0.996
15962 measured reflections
4289 independent reflections
2994 reflections with *I* > 2σ(*I*)
*R*_int_ = 0.048

#### Refinement

*R*[*F* ^2^ > 2σ(*F* ^2^)] = 0.040
*wR*(*F* ^2^) = 0.096
*S* = 1.02
4289 reflections
407 parameters
Only H-atom coordinates refined
Δρ_max_ = 0.24 e Å^−3^
Δρ_min_ = −0.19 e Å^−3^

### 

Data collection: *CrystalClear* (Rigaku, 2004 ▶); cell refinement: *CrystalClear*; data reduction: *CrystalClear*; program(s) used to solve structure: *SHELXS97* (Sheldrick, 2008 ▶); program(s) used to refine structure: *SHELXL97* (Sheldrick, 2008 ▶); molecular graphics: *ORTEPIII* (Burnett & Johnson, 1996 ▶); software used to prepare material for publication: *yadokari-XG* (Wakita, 2005 ▶).

## Supplementary Material

Crystal structure: contains datablocks global, I. DOI: 10.1107/S1600536808001657/om2207sup1.cif

Structure factors: contains datablocks I. DOI: 10.1107/S1600536808001657/om2207Isup2.hkl

Additional supplementary materials:  crystallographic information; 3D view; checkCIF report

## Figures and Tables

**Table 1:** Hydrogen-bond geometry (Å, °)

*D*—H⋯*A*	*D*—H	H⋯*A*	*D*⋯*A*	*D*—H⋯*A*
C18—H15⋯C24^i^	1.03 (2)	2.74 (2)	3.708 (2)	157.0 (15)
C19—H17⋯C2^ii^	1.02 (2)	2.70 (2)	3.568 (2)	142.7 (15)
C23—H25⋯C5^iii^	0.986 (19)	2.82 (2)	3.591 (2)	135.2 (14)

Symmetry codes: (i) 
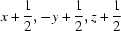
; (ii) 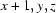; (iii) 
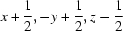
.

**Fig. 1.**
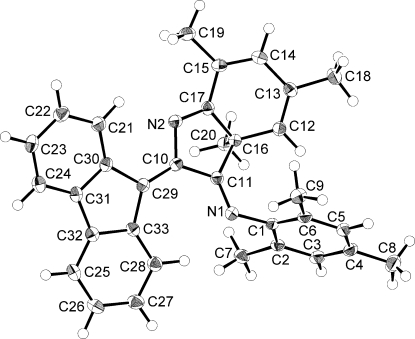
The molecular structure of the title compound, showing the atom-labeling scheme. Displacement ellipsoids are drawn at the 50% probability level.

**Fig. 2.**
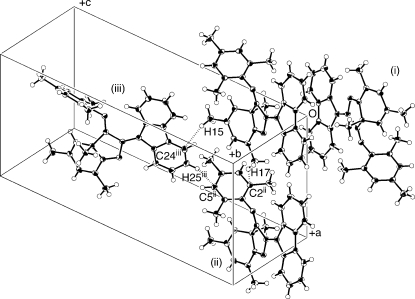
The molecular packing of the title compound. Dashed lines indicate the C–H···π intramolecular contacts [Symmetry codes: (i) -x, -y, -z; (ii) 1 + x, +y, +z; (iii) 1/2 + x, 1/2 - y, 1/2 + z].

## supplementary crystallographic information

### Comment

The structural analysis of 3a*H*-indole, one isomer of indole, has not been achieved due to its instability. On the other hand, the structures of some compounds based on the 3a*H*-indole skeleton, that is, 3,3a-dihydro-2*H*-indole derivertives (Döpp *et al.*, 1985; Miyata *et al.*, 1999; Shimizu *et al.*, 1991) and 3,3a-dihydro-2*H*-indol-2,3-diylidene derivertives (Gerlach *et al.*, 1997; Murakami *et al.*, 1996), have been reported. During our course of studies on the reactivity of a stable stannene (tin–carbon double-bond compound) (Mizuhata *et al.*, 2005), the crystal structure of a new example of 3,3a-dihydro-2*H*-indol-2,3-diylidene derivertives has been revealed.

The title compound was obtained in 29% yield by the reaction of a stannene, Tbt(Mes)Sn?(9-fluorenylidene) (Tbt = 2,4,6-tris[bis(trimethylsilyl)methyl]phenyl; Mes = mesityl), with mesityl isocyanyde. The molecular structure of the title compound is shown in Fig. 1. It was found that the mesityl group is located with *cis* configuration to 3,3a-dihydro-2*H*-indole core with respect to the imine framework. The bond lengths in the 3,3a-dihydro-2*H*-indole core (C10—C17 and N2) are quite similar to those for the related compounds reported previously. However, the C10—C11 bond length [1.501 (3) Å] is longer than those of other 3,3a-dihydro-2*H*-indol-2,3-diylidene derivertives [1.456 (6) Å (Gerlach *et al.*); 1.456 (2) Å (Murakami *et al.*)] and shorter than those of the 3,3a-dihydro-2*H*-indole derivertives [1.553 (3) Å (Döpp *et al.*); 1.566 (3) Å (Miyata *et al.*); 1.565 (5) Å (Shimizu *et al.*)]. The distance between the least squares planes of the center rings of the fluorenylidene groups C29—C30—C31—C32—C33 and C29^i^—C30^i^—C31^i^—C32^i^—C33^i^ (symmetry code: (i) = -*x*, -*y*, -*z*) is 3.510 Å (Fig. 2). The shortest intermolecular contacts were found to be H15—C24^iii^ [2.74 (2) Å], H17—C2^ii^ [2.70 (2) Å], and H25^iii^—C5^ii^ [2.82 (2) Å] (symmetry codes: (ii) 1 + *x*, +*y*, +*z*; (iii) 1/2 + *x*, 1/2 - *y*, 1/2 + *z*).

### Experimental

In a glovebox filled with argon, mesityl isocyanide (7.5 mg, 0.052 mmol) was added to a diethylether (2 ml) solution of Tbt(Mes)Sn?(9-fluorenylidene) [prepared from Tbt(Mes)(9-fluorenyl)SnF (39.3 mg, 0.0403 mmol) and *tert*-butyllithium (0.95 *M* in hexane, 0.043 ml, 0.041 mmol)] at room temperature. The reaction mixture was stirred for 15 h at room temperature. After removal of the solvent, the residue was separated by gel permeation liquid chromatography (eluted with CHCl_3_) to afford the title compound (5.2 mg, 0.0117 mmol, 29%) and MesN?C?(9-fluorenylidene) (3.7 mg, 0.0124 mmol, 31%). Single crystals of the title compound suitable for X-ray crystallographic analysis were obtained as red crystals by slow recrystallization of its benzene solution at room temperature. Physical data: m.p. 411 K (decomposition); ^1^H NMR (300 MHz, CDCl_3_, 298 K): δ 1.21 (s, 3H), 1.67 (s, 3H), 1.91 (s, 3H), 2.23 (s, 3H), 2.28 (s, 3H), 2.34 (s, 3H), 5.22 (s, 1H), 6.25 (s, 1H), 6.88 (s, 1H), 6.96 (s, 1H), 7.13–7.19 (m, 1H), 7.29–7.36 (m, 3H), 7.68–7.70 (m, 2H), 9.06–9.08 (m, 1H), 9.47 (d, ^3^*J* = 8.0 Hz, 1H).

### Refinement

All H atoms were refined with U tied to the bonded C atom: 1.2(*U*_iso_) for C—H and 1.5(*U*_iso_) for CH_3_ groups while all the other atoms were refined anisotropically.

### Crystal data

**Table:**

C_33_H_30_N_2_	*F*_000_ = 968
*M**_r_* = 454.59	*D*_x_ = 1.233 Mg m^−^^3^
Monoclinic, *P*2_1_/*n*	Melting point: 411 K
Hall symbol: -P 2yn	Mo *K*α radiation λ = 0.71069 Å
*a* = 10.2810 (2) Å	θ = 3.4–25.0º
*b* = 11.2727 (3) Å	µ = 0.07 mm^−^^1^
*c* = 21.6598 (5) Å	*T* = 103 (2) K
β = 102.5953 (16)º	Prism, red
*V* = 2449.84 (10) Å^3^	0.20 × 0.20 × 0.05 mm
*Z* = 4	

### Data collection

**Table:**

Rigaku Mercury CCD diffractometer	2994 reflections with *I* > 2σ(*I*)
Radiation source: fine-focus sealed tube	*R*_int_ = 0.048
*T* = 103(2) K	θ_max_ = 25.0º
ω scans	θ_min_ = 3.4º
Absorption correction: multi-scan(REQAB; Jacobson, 1998)	*h* = −12→12
*T*_min_ = 0.986, *T*_max_ = 0.996	*k* = −13→10
15962 measured reflections	*l* = −24→25
4289 independent reflections	

### Refinement

**Table:**

Refinement on *F*^2^	Hydrogen site location: inferred from neighbouring sites
Least-squares matrix: full	Only H-atom coordinates refined
*R*[*F*^2^ > 2σ(*F*^2^)] = 0.040	*w* = 1/[σ^2^(*F*_o_^2^) + (0.0373*P*)^2^ + 0.5999*P*] where *P* = (*F*_o_^2^ + 2*F*_c_^2^)/3
*wR*(*F*^2^) = 0.096	(Δ/σ)_max_ < 0.001
*S* = 1.02	Δρ_max_ = 0.25 e Å^−^^3^
4289 reflections	Δρ_min_ = −0.19 e Å^−^^3^
407 parameters	Extinction correction: SHELXL97 (Sheldrick, 1997), Fc^*^=kFc[1+0.001xFc^2^λ^3^/sin(2θ)]^-1/4^
Primary atom site location: structure-invariant direct methods	Extinction coefficient: 0.0043 (6)
Secondary atom site location: difference Fourier map	

### Special details

**Table:**

Geometry. All e.s.d.'s (except the e.s.d. in the dihedral angle between two l.s. planes) are estimated using the full covariance matrix. The cell e.s.d.'s are taken into account individually in the estimation of e.s.d.'s in distances, angles and torsion angles; correlations between e.s.d.'s in cell parameters are only used when they are defined by crystal symmetry. An approximate (isotropic) treatment of cell e.s.d.'s is used for estimating e.s.d.'s involving l.s. planes.
Refinement. Refinement of *F*^2^ against ALL reflections. The weighted *R*-factor *wR* and goodness of fit *S* are based on *F*^2^, conventional *R*-factors *R* are based on *F*, with *F* set to zero for negative *F*^2^. The threshold expression of *F*^2^ > σ(*F*^2^) is used only for calculating *R*-factors(gt) *etc*. and is not relevant to the choice of reflections for refinement. *R*-factors based on *F*^2^ are statistically about twice as large as those based on *F*, and *R*- factors based on ALL data will be even larger.

### Fractional atomic coordinates and isotropic or equivalent isotropic displacement parameters (Å^2^)

**Table:**

	*x*	*y*	*z*	*U*_iso_*/*U*_eq_	
N1	−0.03226 (14)	0.03480 (13)	0.22158 (7)	0.0217 (4)	
C1	−0.06655 (17)	−0.00324 (16)	0.27895 (8)	0.0202 (4)	
C2	−0.13560 (17)	−0.10987 (16)	0.28032 (8)	0.0206 (4)	
C3	−0.17530 (18)	−0.14060 (17)	0.33593 (9)	0.0236 (4)	
H1	−0.2217 (19)	−0.2182 (17)	0.3372 (8)	0.028*	
C4	−0.15066 (18)	−0.06749 (17)	0.38866 (9)	0.0244 (4)	
C5	−0.08538 (18)	0.03968 (17)	0.38498 (9)	0.0241 (4)	
H2	−0.0656 (18)	0.0949 (17)	0.4239 (9)	0.029*	
C6	−0.04435 (17)	0.07451 (16)	0.33063 (8)	0.0215 (4)	
C7	−0.1717 (2)	−0.18698 (18)	0.22225 (9)	0.0264 (4)	
H3	−0.217 (2)	−0.1430 (18)	0.1834 (10)	0.040*	
H4	−0.235 (2)	−0.2524 (19)	0.2296 (9)	0.040*	
H5	−0.093 (2)	−0.2258 (18)	0.2107 (9)	0.040*	
C8	−0.1956 (2)	−0.1004 (2)	0.44821 (10)	0.0340 (5)	
H6	−0.263 (2)	−0.168 (2)	0.4403 (10)	0.051*	
H7	−0.236 (2)	−0.037 (2)	0.4631 (10)	0.051*	
H8	−0.122 (2)	−0.128 (2)	0.4809 (10)	0.051*	
C9	0.0248 (2)	0.19139 (18)	0.32799 (10)	0.0284 (5)	
H9	0.123 (2)	0.1829 (18)	0.3322 (9)	0.043*	
H10	0.012 (2)	0.2472 (19)	0.3629 (10)	0.043*	
H11	−0.009 (2)	0.2346 (19)	0.2868 (10)	0.043*	
N2	0.26870 (14)	0.06119 (13)	0.16733 (7)	0.0210 (3)	
C10	0.12774 (17)	0.06370 (15)	0.15398 (8)	0.0202 (4)	
C11	0.08304 (17)	0.01780 (15)	0.21100 (8)	0.0195 (4)	
C12	0.24414 (18)	−0.03646 (16)	0.32202 (8)	0.0219 (4)	
H12	0.1716 (18)	−0.0559 (16)	0.3459 (8)	0.026*	
C13	0.37190 (18)	−0.02512 (16)	0.35192 (8)	0.0228 (4)	
C14	0.47680 (18)	−0.00966 (16)	0.31602 (9)	0.0246 (4)	
H13	0.572 (2)	−0.0065 (16)	0.3402 (9)	0.030*	
C15	0.45125 (17)	0.00537 (15)	0.25265 (9)	0.0218 (4)	
C16	0.20669 (17)	−0.04503 (16)	0.25098 (8)	0.0200 (4)	
C17	0.31158 (17)	0.00802 (15)	0.22083 (8)	0.0202 (4)	
C18	0.4151 (2)	−0.03166 (19)	0.42276 (9)	0.0298 (5)	
H14	0.335 (2)	−0.0437 (19)	0.4425 (10)	0.045*	
H15	0.464 (2)	0.0443 (19)	0.4412 (10)	0.045*	
H16	0.479 (2)	−0.102 (2)	0.4354 (9)	0.045*	
C19	0.55426 (19)	0.0312 (2)	0.21488 (10)	0.0278 (5)	
H17	0.647 (2)	0.0295 (18)	0.2433 (10)	0.042*	
H18	0.537 (2)	0.110 (2)	0.1915 (9)	0.042*	
H19	0.549 (2)	−0.0275 (19)	0.1806 (10)	0.042*	
C20	0.1947 (2)	−0.17994 (16)	0.23507 (9)	0.0235 (4)	
H20	0.1700 (19)	−0.1935 (17)	0.1864 (10)	0.035*	
H21	0.127 (2)	−0.2187 (17)	0.2562 (9)	0.035*	
H22	0.284 (2)	−0.2210 (18)	0.2533 (9)	0.035*	
C21	0.25981 (19)	0.18750 (17)	0.05033 (9)	0.0261 (5)	
H23	0.3320 (19)	0.1725 (17)	0.0886 (9)	0.031*	
C22	0.2922 (2)	0.24009 (18)	−0.00252 (9)	0.0287 (5)	
H24	0.387 (2)	0.2675 (17)	−0.0003 (9)	0.034*	
C23	0.1956 (2)	0.26124 (17)	−0.05731 (9)	0.0273 (5)	
H25	0.2183 (19)	0.2987 (17)	−0.0947 (9)	0.033*	
C24	0.0637 (2)	0.23048 (16)	−0.05992 (9)	0.0256 (4)	
H26	−0.005 (2)	0.2484 (16)	−0.0991 (9)	0.031*	
C25	−0.22427 (19)	0.14758 (17)	−0.03726 (9)	0.0256 (4)	
H27	−0.2330 (18)	0.1752 (17)	−0.0804 (9)	0.031*	
C26	−0.3349 (2)	0.11042 (17)	−0.01557 (9)	0.0281 (5)	
H28	−0.425 (2)	0.1090 (17)	−0.0438 (9)	0.034*	
C27	−0.3212 (2)	0.07354 (17)	0.04646 (9)	0.0280 (5)	
H29	−0.398 (2)	0.0512 (17)	0.0631 (9)	0.034*	
C28	−0.19683 (18)	0.07043 (16)	0.08820 (9)	0.0250 (4)	
H30	−0.1866 (18)	0.0447 (16)	0.1337 (9)	0.030*	
C29	0.06016 (17)	0.10611 (16)	0.09684 (8)	0.0201 (4)	
C30	0.12739 (18)	0.15654 (15)	0.04819 (8)	0.0205 (4)	
C31	0.03035 (18)	0.17914 (15)	−0.00726 (8)	0.0209 (4)	
C32	−0.10020 (18)	0.14522 (15)	0.00361 (8)	0.0214 (4)	
C33	−0.08408 (18)	0.10508 (15)	0.06673 (8)	0.0205 (4)	

### Atomic displacement parameters (Å^2^)

**Table:**

	*U*^11^	*U*^22^	*U*^33^	*U*^12^	*U*^13^	*U*^23^
N1	0.0207 (8)	0.0246 (9)	0.0202 (8)	0.0006 (7)	0.0055 (7)	0.0002 (7)
C1	0.0153 (9)	0.0266 (10)	0.0188 (10)	0.0045 (8)	0.0039 (8)	0.0037 (8)
C2	0.0159 (9)	0.0228 (10)	0.0225 (10)	0.0027 (8)	0.0027 (8)	0.0005 (8)
C3	0.0194 (10)	0.0238 (10)	0.0282 (11)	0.0026 (8)	0.0066 (8)	0.0034 (9)
C4	0.0187 (10)	0.0302 (11)	0.0259 (10)	0.0051 (8)	0.0084 (8)	0.0035 (9)
C5	0.0191 (10)	0.0311 (11)	0.0222 (10)	0.0041 (8)	0.0048 (8)	−0.0026 (9)
C6	0.0144 (9)	0.0250 (10)	0.0249 (10)	0.0027 (7)	0.0040 (8)	−0.0009 (8)
C7	0.0235 (11)	0.0276 (11)	0.0269 (11)	−0.0028 (9)	0.0030 (9)	−0.0026 (9)
C8	0.0338 (13)	0.0398 (13)	0.0313 (12)	0.0034 (10)	0.0136 (10)	0.0056 (10)
C9	0.0270 (11)	0.0265 (11)	0.0329 (12)	−0.0024 (9)	0.0090 (10)	−0.0051 (9)
N2	0.0184 (8)	0.0226 (8)	0.0222 (8)	0.0006 (7)	0.0046 (7)	0.0005 (7)
C10	0.0198 (9)	0.0196 (9)	0.0221 (10)	0.0010 (8)	0.0065 (8)	−0.0003 (8)
C11	0.0197 (10)	0.0197 (9)	0.0188 (9)	−0.0005 (7)	0.0035 (8)	−0.0025 (8)
C12	0.0231 (10)	0.0227 (10)	0.0203 (10)	0.0029 (8)	0.0054 (8)	0.0010 (8)
C13	0.0239 (10)	0.0219 (10)	0.0213 (10)	0.0021 (8)	0.0018 (8)	0.0010 (8)
C14	0.0197 (10)	0.0243 (10)	0.0275 (11)	−0.0006 (8)	0.0003 (9)	0.0008 (8)
C15	0.0190 (9)	0.0192 (10)	0.0268 (10)	−0.0002 (8)	0.0040 (8)	0.0004 (8)
C16	0.0175 (9)	0.0230 (10)	0.0192 (9)	0.0004 (7)	0.0035 (8)	−0.0003 (8)
C17	0.0221 (10)	0.0194 (10)	0.0194 (10)	0.0012 (8)	0.0054 (8)	−0.0018 (8)
C18	0.0319 (12)	0.0327 (12)	0.0228 (11)	−0.0038 (10)	0.0015 (9)	0.0022 (9)
C19	0.0201 (11)	0.0323 (12)	0.0312 (11)	−0.0001 (9)	0.0060 (9)	0.0026 (10)
C20	0.0224 (10)	0.0206 (10)	0.0271 (11)	0.0018 (8)	0.0041 (9)	0.0022 (8)
C21	0.0252 (11)	0.0282 (11)	0.0249 (11)	0.0024 (8)	0.0059 (9)	0.0046 (9)
C22	0.0279 (11)	0.0289 (11)	0.0312 (11)	0.0008 (9)	0.0107 (9)	0.0048 (9)
C23	0.0360 (12)	0.0247 (11)	0.0233 (11)	−0.0021 (9)	0.0114 (9)	0.0022 (8)
C24	0.0327 (11)	0.0220 (10)	0.0215 (10)	0.0007 (9)	0.0046 (9)	−0.0004 (8)
C25	0.0307 (11)	0.0239 (10)	0.0199 (10)	0.0013 (9)	0.0006 (9)	0.0008 (8)
C26	0.0238 (11)	0.0269 (11)	0.0294 (11)	−0.0023 (9)	−0.0031 (9)	0.0020 (9)
C27	0.0231 (11)	0.0282 (11)	0.0319 (12)	−0.0042 (9)	0.0042 (9)	0.0049 (9)
C28	0.0249 (11)	0.0248 (10)	0.0245 (10)	−0.0002 (8)	0.0039 (9)	0.0027 (8)
C29	0.0216 (10)	0.0184 (9)	0.0206 (10)	0.0007 (8)	0.0055 (8)	−0.0017 (8)
C30	0.0242 (10)	0.0175 (9)	0.0211 (10)	0.0019 (8)	0.0076 (8)	−0.0008 (8)
C31	0.0271 (10)	0.0167 (10)	0.0191 (10)	0.0021 (8)	0.0053 (8)	−0.0016 (7)
C32	0.0253 (10)	0.0175 (9)	0.0211 (10)	0.0025 (8)	0.0041 (8)	−0.0027 (8)
C33	0.0220 (10)	0.0179 (9)	0.0205 (9)	0.0028 (8)	0.0022 (8)	0.0002 (8)

### Geometric parameters (Å, °)

**Table:**

N1—C11	1.270 (2)	C15—C19	1.501 (3)
N1—C1	1.429 (2)	C16—C17	1.501 (2)
C1—C2	1.399 (2)	C16—C20	1.558 (3)
C1—C6	1.401 (2)	C18—H14	1.02 (2)
C2—C3	1.397 (2)	C18—H15	1.03 (2)
C2—C7	1.507 (3)	C18—H16	1.02 (2)
C3—C4	1.386 (3)	C19—H17	1.02 (2)
C3—H1	1.000 (19)	C19—H18	1.02 (2)
C4—C5	1.393 (3)	C19—H19	0.99 (2)
C4—C8	1.508 (3)	C20—H20	1.04 (2)
C5—C6	1.391 (3)	C20—H21	1.01 (2)
C5—H2	1.033 (19)	C20—H22	1.03 (2)
C6—C9	1.504 (3)	C21—C22	1.392 (3)
C7—H3	1.00 (2)	C21—C30	1.396 (2)
C7—H4	1.02 (2)	C21—H23	1.00 (2)
C7—H5	1.00 (2)	C22—C23	1.392 (3)
C8—H6	1.02 (2)	C22—H24	1.01 (2)
C8—H7	0.93 (2)	C23—C24	1.389 (3)
C8—H8	0.97 (2)	C23—H25	0.986 (19)
C9—H9	1.00 (2)	C24—C31	1.387 (3)
C9—H10	1.02 (2)	C24—H26	1.00 (2)
C9—H11	1.01 (2)	C25—C32	1.385 (3)
N2—C17	1.294 (2)	C25—C26	1.387 (3)
N2—C10	1.415 (2)	C25—H27	0.970 (19)
C10—C29	1.367 (2)	C26—C27	1.384 (3)
C10—C11	1.501 (2)	C26—H28	0.99 (2)
C11—C16	1.545 (2)	C27—C28	1.396 (3)
C12—C13	1.338 (3)	C27—H29	0.969 (19)
C12—C16	1.506 (2)	C28—C33	1.396 (2)
C12—H12	1.020 (18)	C28—H30	1.010 (18)
C13—C14	1.471 (3)	C29—C33	1.484 (2)
C13—C18	1.503 (3)	C29—C30	1.493 (2)
C14—C15	1.351 (2)	C30—C31	1.407 (2)
C14—H13	1.01 (2)	C31—C32	1.463 (2)
C15—C17	1.451 (2)	C32—C33	1.415 (2)
			
C11—N1—C1	121.90 (15)	C11—C16—C20	107.90 (14)
C2—C1—C6	121.03 (16)	N2—C17—C15	122.92 (16)
C2—C1—N1	119.90 (16)	N2—C17—C16	115.94 (15)
C6—C1—N1	118.61 (16)	C15—C17—C16	121.02 (15)
C3—C2—C1	118.44 (17)	C13—C18—H14	110.5 (12)
C3—C2—C7	120.75 (17)	C13—C18—H15	111.5 (11)
C1—C2—C7	120.76 (16)	H14—C18—H15	108.6 (16)
C4—C3—C2	121.85 (18)	C13—C18—H16	109.9 (11)
C4—C3—H1	120.0 (10)	H14—C18—H16	108.2 (17)
C2—C3—H1	118.1 (10)	H15—C18—H16	108.1 (17)
C3—C4—C5	118.19 (17)	C15—C19—H17	110.4 (11)
C3—C4—C8	121.55 (19)	C15—C19—H18	111.8 (11)
C5—C4—C8	120.25 (18)	H17—C19—H18	111.0 (16)
C6—C5—C4	122.15 (17)	C15—C19—H19	110.7 (12)
C6—C5—H2	118.7 (10)	H17—C19—H19	109.3 (17)
C4—C5—H2	119.2 (10)	H18—C19—H19	103.5 (16)
C5—C6—C1	118.23 (17)	C16—C20—H20	111.0 (11)
C5—C6—C9	120.84 (17)	C16—C20—H21	110.5 (11)
C1—C6—C9	120.92 (16)	H20—C20—H21	110.9 (16)
C2—C7—H3	113.6 (12)	C16—C20—H22	109.5 (11)
C2—C7—H4	109.7 (11)	H20—C20—H22	108.9 (15)
H3—C7—H4	107.3 (16)	H21—C20—H22	106.0 (15)
C2—C7—H5	113.4 (12)	C22—C21—C30	119.02 (18)
H3—C7—H5	105.1 (16)	C22—C21—H23	119.2 (11)
H4—C7—H5	107.4 (16)	C30—C21—H23	121.8 (11)
C4—C8—H6	111.4 (12)	C23—C22—C21	121.32 (19)
C4—C8—H7	110.8 (14)	C23—C22—H24	119.2 (11)
H6—C8—H7	106.9 (18)	C21—C22—H24	119.3 (11)
C4—C8—H8	111.7 (13)	C24—C23—C22	120.05 (18)
H6—C8—H8	106.2 (18)	C24—C23—H25	118.5 (11)
H7—C8—H8	109.6 (19)	C22—C23—H25	121.4 (11)
C6—C9—H9	112.9 (12)	C31—C24—C23	119.00 (18)
C6—C9—H10	112.0 (11)	C31—C24—H26	122.0 (11)
H9—C9—H10	106.7 (17)	C23—C24—H26	119.0 (11)
C6—C9—H11	112.4 (12)	C32—C25—C26	119.13 (18)
H9—C9—H11	105.9 (16)	C32—C25—H27	120.0 (11)
H10—C9—H11	106.4 (17)	C26—C25—H27	120.8 (11)
C17—N2—C10	109.04 (14)	C27—C26—C25	120.13 (18)
C29—C10—N2	119.21 (15)	C27—C26—H28	118.6 (11)
C29—C10—C11	132.80 (16)	C25—C26—H28	121.2 (11)
N2—C10—C11	107.98 (14)	C26—C27—C28	121.43 (19)
N1—C11—C10	124.19 (16)	C26—C27—H29	121.3 (12)
N1—C11—C16	130.86 (15)	C28—C27—H29	117.3 (12)
C10—C11—C16	104.90 (14)	C33—C28—C27	119.15 (18)
C13—C12—C16	120.40 (16)	C33—C28—H30	119.2 (11)
C13—C12—H12	121.6 (10)	C27—C28—H30	121.7 (11)
C16—C12—H12	116.9 (10)	C10—C29—C33	131.08 (16)
C12—C13—C14	120.73 (17)	C10—C29—C30	123.37 (16)
C12—C13—C18	122.00 (17)	C33—C29—C30	105.36 (14)
C14—C13—C18	117.24 (17)	C21—C30—C31	119.19 (16)
C15—C14—C13	123.33 (17)	C21—C30—C29	132.06 (16)
C15—C14—H13	118.5 (10)	C31—C30—C29	108.67 (15)
C13—C14—H13	118.1 (10)	C24—C31—C30	121.41 (17)
C14—C15—C17	115.99 (16)	C24—C31—C32	129.88 (17)
C14—C15—C19	125.04 (17)	C30—C31—C32	108.65 (15)
C17—C15—C19	118.67 (16)	C25—C32—C33	121.41 (17)
C17—C16—C12	111.78 (15)	C25—C32—C31	129.88 (17)
C17—C16—C11	98.91 (13)	C33—C32—C31	108.71 (15)
C12—C16—C11	122.63 (14)	C28—C33—C32	118.73 (16)
C17—C16—C20	108.81 (14)	C28—C33—C29	132.74 (16)
C12—C16—C20	106.20 (14)	C32—C33—C29	108.50 (15)
			
C11—N1—C1—C2	−98.5 (2)	C19—C15—C17—C16	164.92 (17)
C11—N1—C1—C6	89.2 (2)	C12—C16—C17—N2	−145.07 (16)
C6—C1—C2—C3	−3.7 (3)	C11—C16—C17—N2	−14.51 (19)
N1—C1—C2—C3	−175.83 (15)	C20—C16—C17—N2	97.96 (18)
C6—C1—C2—C7	173.55 (17)	C12—C16—C17—C15	31.1 (2)
N1—C1—C2—C7	1.4 (2)	C11—C16—C17—C15	161.68 (15)
C1—C2—C3—C4	1.4 (3)	C20—C16—C17—C15	−85.9 (2)
C7—C2—C3—C4	−175.85 (17)	C30—C21—C22—C23	−0.6 (3)
C2—C3—C4—C5	0.6 (3)	C21—C22—C23—C24	0.4 (3)
C2—C3—C4—C8	179.11 (18)	C22—C23—C24—C31	0.2 (3)
C3—C4—C5—C6	−0.3 (3)	C32—C25—C26—C27	1.1 (3)
C8—C4—C5—C6	−178.86 (18)	C25—C26—C27—C28	−1.3 (3)
C4—C5—C6—C1	−1.9 (3)	C26—C27—C28—C33	−0.1 (3)
C4—C5—C6—C9	179.43 (18)	N2—C10—C29—C33	170.95 (17)
C2—C1—C6—C5	4.0 (3)	C11—C10—C29—C33	−10.7 (3)
N1—C1—C6—C5	176.18 (15)	N2—C10—C29—C30	−3.3 (3)
C2—C1—C6—C9	−177.41 (17)	C11—C10—C29—C30	175.04 (17)
N1—C1—C6—C9	−5.2 (2)	C22—C21—C30—C31	0.1 (3)
C17—N2—C10—C29	−174.03 (16)	C22—C21—C30—C29	−176.18 (18)
C17—N2—C10—C11	7.24 (19)	C10—C29—C30—C21	−10.0 (3)
C1—N1—C11—C10	−175.21 (16)	C33—C29—C30—C21	174.43 (19)
C1—N1—C11—C16	1.9 (3)	C10—C29—C30—C31	173.40 (17)
C29—C10—C11—N1	−16.7 (3)	C33—C29—C30—C31	−2.11 (19)
N2—C10—C11—N1	161.79 (16)	C23—C24—C31—C30	−0.7 (3)
C29—C10—C11—C16	165.60 (19)	C23—C24—C31—C32	176.07 (18)
N2—C10—C11—C16	−15.91 (18)	C21—C30—C31—C24	0.6 (3)
C16—C12—C13—C14	5.7 (3)	C29—C30—C31—C24	177.65 (16)
C16—C12—C13—C18	−172.16 (16)	C21—C30—C31—C32	−176.83 (16)
C12—C13—C14—C15	6.5 (3)	C29—C30—C31—C32	0.2 (2)
C18—C13—C14—C15	−175.49 (18)	C26—C25—C32—C33	0.4 (3)
C13—C14—C15—C17	1.2 (3)	C26—C25—C32—C31	−179.99 (18)
C13—C14—C15—C19	174.80 (18)	C24—C31—C32—C25	5.1 (3)
C13—C12—C16—C17	−22.9 (2)	C30—C31—C32—C25	−177.79 (19)
C13—C12—C16—C11	−139.88 (18)	C24—C31—C32—C33	−175.26 (18)
C13—C12—C16—C20	95.6 (2)	C30—C31—C32—C33	1.9 (2)
N1—C11—C16—C17	−160.50 (19)	C27—C28—C33—C32	1.6 (3)
C10—C11—C16—C17	16.99 (17)	C27—C28—C33—C29	−176.13 (18)
N1—C11—C16—C12	−37.4 (3)	C25—C32—C33—C28	−1.8 (3)
C10—C11—C16—C12	140.10 (16)	C31—C32—C33—C28	178.55 (16)
N1—C11—C16—C20	86.3 (2)	C25—C32—C33—C29	176.49 (17)
C10—C11—C16—C20	−96.19 (16)	C31—C32—C33—C29	−3.20 (19)
C10—N2—C17—C15	−170.90 (16)	C10—C29—C33—C28	6.1 (3)
C10—N2—C17—C16	5.2 (2)	C30—C29—C33—C28	−178.83 (19)
C14—C15—C17—N2	154.85 (17)	C10—C29—C33—C32	−171.78 (19)
C19—C15—C17—N2	−19.2 (3)	C30—C29—C33—C32	3.26 (19)
C14—C15—C17—C16	−21.1 (2)		

### Hydrogen-bond geometry (Å, °)

**Table:**

*D*—H···*A*	*D*—H	H···*A*	*D*···*A*	*D*—H···*A*
C18—H15···C24^i^	1.03 (2)	2.74 (2)	3.708 (2)	157.0 (15)
C19—H17···C2^ii^	1.02 (2)	2.70 (2)	3.568 (2)	142.7 (15)
C23—H25···C5^iii^	0.986 (19)	2.82 (2)	3.591 (2)	135.2 (14)

Symmetry codes: (i) *x*+1/2, −*y*+1/2, *z*+1/2; (ii) *x*+1, *y*, *z*; (iii) *x*+1/2, −*y*+1/2, *z*−1/2.
